# Introducing the *Journal of Medical Extended Reality*

**DOI:** 10.1089/jmxr.2024.28999.editorial

**Published:** 2024-01-25

**Authors:** Haipeng Zhang, Brennan Spiegel

**Affiliations:** ^1^ Office of Healthcare Innovation and Learning, United States Department of Veteran Affairs, Washington, District of Columbia, USA.; ^2^ Department of Psychosocial Oncology and Palliative Care, Dana-Farber Cancer Institute, Boston, Massachusetts, USA.; ^3^ Division of Health Services Research, Department of Medicine, Cedars-Sinai, Los Angeles, California, USA.

The first decade of the 21st century witnessed the ascent of advanced personal computing devices, marking a paradigm shift that has transformed every aspect of our daily lives, including the experience and delivery of health care. Extended reality (XR)—encompassing virtual reality (VR), augmented reality, mixed reality, and other forms of spatial computing—is on course to affect a comparable widespread impact on society and the health care sector.

The origins of medical extended reality (MXR) are rooted in the early explorations of VR during the 1980s and 1990s, which were initially applied in psychological therapy and simulation-based training.^1,2^ As technological capabilities have evolved, the spectrum of MXR applications has expanded substantially, leading to its recognition and validation by regulatory authorities such as the U.S. Food and Drug Administration (FDA).^3^ This formal recognition is a significant hallmark of progress for MXR, signaling not only its legitimacy but also encouraging further innovation and implementation of these technologies into health care delivery.

Since the early 2010s, the applications of XR in health care have surged, propelled by the advent of accessible development platforms that have lowered barriers to innovation in spatial computing. This democratization has not only revitalized the XR industry but also spurred significant growth across various sectors, including health care. Coupled with exponential advancements in computational power and artificial intelligence (AI), these developments are setting the stage for unprecedented progress in how we interact with digital environments and how health care can be augmented by these cutting-edge technologies.

The expanding compendium of MXR research now spans an extensive range of subjects. It delves into therapeutic applications such as pain mitigation,^4,5^ rehabilitative strategies,^6,7^ and psychological interventions,^8,9^ extends into procedural skill enhancement^10,11^ and medical pedagogy,^12^ and encompasses the ethical considerations and societal reception of immersive technologies. It also explores the foundational technologies of MXR, combining AI with spatial computing to forge new frontiers.^13,14^ This assortment of topics not only illustrates the adaptability of MXR to meet diverse health care challenges but also highlights its broad influence across the spectrum of medical disciplines, underscoring the multidisciplinary nature of its evolution and implementation.

In August of 2022, the American Medical Extended Reality Association (AMXRA) was established as a foundational organization dedicated to supporting MXR through the creation of a professional society dedicated to this burgeoning field. The mission of AMXRA is to advance the science and practice of MXR through promoting best practices in care delivery, scientific investigation, innovation, education, advocacy, and community. In partnership with the society's publishing partner, Mary Ann Liebert, one of AMXRA's first major initiatives was the creation of a peer-reviewed journal entirely dedicated to MXR—the *Journal of Medical Extended Reality*.

The journal stands at the forefront of the field of MXR, an interdisciplinary science focused on the role of XR technologies in health care. With the rapid advances in XR hardware and software, it is vital to examine how XR technologies can promote human health and well-being at scale. As the journal of record for MXR and the official journal of the AMXRA, the *Journal of Medical Extended Reality* serves as a platform for researchers, practitioners, and innovators across disciplines to explore the multifaceted role of XR in health care and a foundational core for the MXR field.

The establishment of the journal is foundational to defining the current and future states of MXR, the science that supports MXR use, and detailing breakthroughs in MXR. We believe that MXR will become a distinct field of medicine in the 21st century and that AMXRA, along with its flagship journal, will help elevate this movement by featuring cutting edge international research within the field of MXR.

We are excited to share the inaugural collection of articles for the journal. Among them is the first of many planned AMXRA guidelines, titled “What is Medical Extended Reality? A Taxonomy Defining the Current Breadth and Depth of an Evolving Field.”^15^ In this guidance document, a panel of AMXRA members, including members of the editorial advisory board for the journal, introduce a comprehensive taxonomy for MXR, developed through a multidisciplinary and international collaboration among experts.

By seeking to standardize terminology, categorize existing work, and provide a structured framework for future research and development in MXR, the guideline offers a framework for researchers, clinicians, funders, academic publishers, and regulators, facilitating clearer communication and categorization in this field. As MXR grows, we hope this AMXRA document will be helpful in guiding the field's development and ensuring a cohesive understanding of its multifaceted nature.

It is an honor and privilege to help launch this new journal in collaboration with an incredible editorial board hailing from around the world. In selecting the inaugural board, we sought to engage a diverse group of editors based on their publications in the field, foundational contributions to MXR, international perspectives, and multidisciplinary expertise. Without their tireless dedication to the field, and to the journal, we would not be able to write these words. We look forward to expanding the publication channels to accommodate the burgeoning number of MXR articles, and look forward to a collegial and collaborative future for the journal that supports the growing worldwide MXR community and the patients they serve.

## Abbreviations Used

AI: artificial intelligence
AMXRA: Amerxican Medical Extended Reality Association
MXR: medical extended reality
VR: virtual reality
XR: extended reality

## References

[1] Greenleaf W, Roberts LaVonne, et al. Applied virtual reality in healthcare: Case studies and perspectives. Cool Blue Media; 2021.

[2] Spiegel BS. VRx: How Immersive Therapeutics Will Revolutionize Medicine. Basic Books: New York, NY; 2020.

[3] United States Food and Drug Administration (FDA). Medical Extended Reality Program: Research on Medical Extended Reality-Based Medical Devices. U.S. Food and Drug Administration. 2023. Updated March 24. Available from: https://www.fda.gov/medical-devices/medical-device-regulatory-science-research-programs-conducted-osel/medical-extended-reality-program-research-medical-extended-reality-based-medical-devices [Last accessed: April 17, 2023].

[4] Lier EJ, de Vries M, Steggink EM, et al. Effect modifiers of virtual reality in pain management: A systematic review and meta-regression analysis. Pain 2023;164:1685–1665. 10.1097/j.pain.0000000000002883 PMC10348651 36943251

[5] Goudman L, Jansen J, Billot M, et al. Virtual reality applications in chronic pain management: Systematic review and meta-analysis. JMIR Serious Games 2022;10:e34402. 35536641 10.2196/34402 PMC9131143

[6] Laver KE, Lange B, George S, et al. Virtual reality for stroke rehabilitation. Cochrane Database Syst Rev 2017;11:CD008349. 29156493 10.1002/14651858.CD008349.pub4 PMC6485957

[7] Rutkowski S, Kiper P, Cacciante L, et al. Use of virtual reality-based training in different fields of rehabilitation: A systematic review and meta-analysis. J Rehabil Med 2020;52:jrm00121. 33073855 10.2340/16501977-2755

[8] Carl E, Stein AT, Levihn-Coon A, et al. Virtual reality exposure therapy for anxiety and related disorders: A meta-analysis of randomized controlled trials. J Anxiety Disord 2019;61:27–36. 30287083 10.1016/j.janxdis.2018.08.003

[9] Van Loenen I, Scholten W, Muntingh A, et al. The effectiveness of virtual reality exposure-based cognitive behavioral therapy for severe anxiety disorders, obsessive-compultive disorder, and posttraumatic stress disorder: Meta-analysis. J Med Internet Res 2022;24:e26736. 35142632 10.2196/26736 PMC8874794

[10] Nagendran M, Gurusamy KS, Aggarwal R, et al. Virtual reality training for surgical trainees in laparoscopic surgery. Cochrane Databsae Syst Rev 2013(8):CD006575. 10.1002/14651858.CD006575.pub3 PMC7388923 23980026

[11] Laskay NMB, George JA, Knowlin L, et al. Optimizing surgical performance using preoperative virtual reality planning: A systematic review. World J Surg 2023;47:2367–2377. 37204439 10.1007/s00268-023-07064-8

[12] Barteit S, Lanfermann L, Barnighausen T, et al. Augmented, mixed, and virtual reality-based head-mounted devices for medical education: Systematic review. JMIR Serious Games 2021;9:e29080. 34255668 10.2196/29080 PMC8299342

[13] Harmon J, Pitt V, Summos P, et al. Use of artificial intelligence and virtual reality within clinical simulation for nursing pain education: A scoping review. Nurse Educ Today 2021;97:104700. 33341064 10.1016/j.nedt.2020.104700

[14] Antel R, Abbasgholizadeh-Rahimi S, Guadagno E, et al. The use of artificial intelligence and virtual reality in doctor-patient risk communication: A scoping review. Patient Educ Couns 2022;105:3038–3050. 35725526 10.1016/j.pec.2022.06.006

[15] Spiegel BMR, Rizzo A, Persky S, et al. AMXRA guideline: What is medical extended reality? A taxonomy defining the current breadth and depth of an evolving field. J Med Extended Reality 2024; doi: 10.1089/JMXR.2023.0012 PMC10945763 38505474

